# Objective and perceived smartphone use volume, addiction risk, and deprivation feelings

**DOI:** 10.1016/j.abrep.2026.100750

**Published:** 2026-09-21

**Authors:** Attila Szabo, Róbert Járai, Norbert Tabi, Dorina Montvai, Celal Bulgay, Robert Podstawski, Angéla Somogyi

**Affiliations:** a Faculty of Health and Sport Sciences, Széchenyi István University, Győr, Hungary; b Institute of Health Promotion and Sport Sciences, ELTE Eötvös Loránd University, Budapest, Hungary; c Faculty of Sport Sciences, Bingöl University, Bingöl, Türkiye; d Department of Physiotherapy, University of Warmia and Mazury, Olsztyn, Poland

**Keywords:** Risk of smartphone addiction, Screen time, Objective smartphone use, Perceived smartphone use, Withdrawal symptoms

## Abstract

**Background:**

Research on the risk of smartphone addiction (RSA) relies on self-reports, despite evidence that subjective and objective measures are unrelated. This study examined relationships among recorded iPhone use, perceived use, app patterns, smartphone deprivation feelings (SDF), and RSA.

**Methods:**

One hundred iPhone users (M_age_ = 24.37 ± 7.78; 38 males) provided screenshots of iOS *Screen Time* data collected over eight days. We combined indicators of iPhone use across app categories into hedonic and utilitarian indices and assessed RSA using the Smartphone Application Based Addiction Scale (SABAS) and SDF using a Smartphone Deprivation Inventory.

**Results:**

Based on the SABAS, 29% of participants were at high risk of RSA, which correlated with SDF, hedonic use, and perceived use. Objective use showed a weak association with RSA (*r* = 0.20, 95% CI [−0.001, 0.38]). Regression revealed that SDF and perceived use were independently associated with RSA, explaining 33.8% of the variance, while hedonic use, age, and gender added no additional variance. Perceived use mediated the link between objective iPhone use and RSA. RSA was more strongly associated with SDF than with objective use. Nearly 80% of use was hedonic, with over 50% related to social media.

**Conclusions:**

These findings suggest that self-appraised smartphone use and SDF are more closely related to questionnaire-assessed RSA than to objectively measured volume. Since RSA, perceived use, and SDF were all self-reported, the results do not imply that actual use volume is unimportant. Instead, they highlight the importance of subjective appraisal and deprivation-related experiences in RSA assessments.

## Highlights

• Smartphone addiction risk was more closely linked to perceived than objectively recorded iPhone use.
• Deprivation feelings were the strongest predictor of smartphone addiction risk.
• Objective screen time predicted addiction risk indirectly through perceived use.
• Social media applications accounted for over half of the recorded iPhone use.
• Addiction-risk scores related more strongly to perceived use and deprivation than logged screen time.

## 1 Introduction

Smartphone-based application addiction has emerged as a significant social and public health concern worldwide (Das & Chaliha, 2025; Shahjehan et al., 2021). The ubiquity of smartphones in daily life has transformed how individuals communicate, work, and access entertainment (Ahmead et al., 2025). Beyond facilitating everyday activities (Roos & Wrzus, 2022), these devices can provide immediate gratification through social networking, gaming, and entertainment applications, thereby fostering compulsive use. Problematic smartphone use (PSU) is associated with decreased academic performance, impaired social relationships, and psychological distress (Billieux et al., 2015).

The terms problematic smartphone use (PSU) and smartphone addiction are often used interchangeably, although notable conceptual distinctions have been proposed. Smartphone addiction is commonly described as a maladaptive pattern of use characterized by loss of control, tolerance, withdrawal, and negative consequences, and is frequently framed within the broader category of behavioral addictions (Billieux, 2012; Griffiths, 2005). In contrast, PSU has been introduced as a more conservative umbrella term referring to excessive or poorly regulated smartphone use associated with functional impairment, without implying a clinical addiction diagnosis (Panova & Carbonell, 2018). Neither construct is currently recognized as a formal disorder in medical diagnostic reference manuals such as the Diagnostic and Statistical Manual of Mental Disorders (DSM-5; American Psychiatric Association, 2013), contributing to ongoing terminological inconsistency in the literature (Panova & Carbonell, 2018). To maintain terminological consistency, the present study uses the term risk of smartphone addiction (RSA) throughout to refer to elevated scores on a brief self-report screening instrument; the terms smartphone addiction and PSU are used only when reproducing the constructs as labeled in the cited literature.

In the absence of a clinical diagnosis, questionnaire scores alone cannot establish smartphone addiction. Instead, they indicate only a risk factor, and several authors use RSA to denote this issue (e.g., Cocoradă et al., 2018; Osorio-Molina et al., 2021; Park, 2019). The proportion of individuals with elevated RSA varies widely across populations, with university students being a particularly vulnerable group (Peng et al., 2023). Across 82 multinational studies comprising 147,319 participants, Meng et al. (2022) found a pooled prevalence of 26.99% for smartphone addiction. Thus, understanding the correlates of RSA has become increasingly urgent as mobile devices continue to spread globally.

Research on smartphone addiction has grown rapidly over the past decade (Wu & Chou, 2023). Numerous psychometric instruments have been developed to assess PSU and RSA across diverse cultural contexts (Csibi et al., 2018; Meng et al., 2022). Cross-sectional studies have investigated psychological predictors, usage patterns, and intervention strategies for PSU and RSA (Pirwani and Szabo, 2024, Pirwani and Szabo, 2025). Cross-cultural validation studies have designed measurement tools in multiple languages, facilitating international research collaboration (Vujić et al., 2023). Despite the proliferation of research, methodological inconsistencies and conceptual ambiguities complicate the synthesis of findings. The rapid evolution of smartphone technology and applications presents ongoing challenges for researchers seeking to accurately capture the phenomenon (McDuff et al., 2024).

Hypothetically, greater smartphone use is expected to be associated with higher RSA, but limited evidence supports this conjecture (Tangmunkongvorakul et al., 2020). However, empirical findings on this relationship are mixed (Hitcham et al., 2023; Molaib et al., 2025). Research reveals that psychological factors such as smartphone deprivation feelings (SDF), anxiety, and loss of control may be more central to addiction than just usage volume (Eide et al., 2018; Li et al., 2024). The line between excessive use and true addiction remains poorly understood (Billieux, 2012).

Beyond usage volume, motivation for use may also matter. In technology-acceptance research, a distinction exists between *hedonic* and *utilitarian* systems; hedonic use involves pleasure and entertainment, while utilitarian use is goal-oriented, focusing on tasks and information (van der Heijden, 2004). On smartphones, hedonic activities may include social media and gaming, whereas utilitarian functions cover productivity and health (Vujić & Szabo, 2022). This distinction matters for RSA because hedonic use, driven by reward, may lead to compulsive-like behaviors linked to problematic technology use (Brand et al., 2016; Elhai et al., 2019). Vujić and Szabo (2022) found that hedonic use predicted RSA, but utilitarian use did not. Yet, few studies examine these use types in relation to RSA, especially using logged application data, which this study aims to address.

Little research has measured objective smartphone usage; only approximately 10% of studies use logged behavioral data (James et al., 2023), whereas most rely exclusively on self-reported usage estimates. Recent evidence indicates poor to moderate agreement between subjective and objective usage data, raising concerns about measurement validity in addiction research (Molaib et al., 2025). Therefore, objective data tracking is needed. One way to obtain such objective records without installing third-party tracking software is to rely on the logging functions built into the smartphone's operating system, as described next.

Apple's *Screen Time* feature, introduced with iOS 12 in 2018, passively logs active screen time at the operating-system level and reports use durations for individual applications and for predefined application categories (e.g., Social Networking, Entertainment, Games, Productivity & Finance) derived from App Store metadata (Apple Inc., 2026). In the present study, this feature was used solely to obtain each participant's total and daily use duration and the time spent within each application category. Validation research indicates that iOS *Screen Time* provides objective measurements suitable for research applications, correlating strongly (*r* = 0.88, 95% CI: 0.84–0.91) with an independent research-based tracking application (Kristensen et al., 2022).

A recent study by Hitcham et al. (2023) used *Screen Time* data and found that RSA is more closely related to perceived rather than objectively logged use, with self-reported use mediating more than half of the relationship between actual usage and RSA across two time points. Their findings indicated that participants who systematically overestimated their iPhone use reported higher RSA, independent of actual usage volume, and that perceived use showed broad associations with RSA even when objective and subjective measures were incongruent. However, the study was limited by its focus on total screen time, reliance on aggregate addiction scores, and the absence of SDF measures.

The present study, in addition to attempting to replicate Hitcham et al.'s (2023) mediation results, extends their research by categorizing *hedonic* and *utilitarian* usage patterns (Vujić & Szabo, 2022), examining component-level addiction symptoms, and directly assessing SDF, thereby enabling a more detailed examination of the correlates of RSA beyond mere usage volume. At an exploratory level, we expected that objective, subjective, and hedonic iPhone use, along with SDF, would account for a significant proportion of variance in RSA, and that time spent with applications listed in the social networking category by *Screen Time*, as a unique component of hedonic use, would be most closely linked to RSA scores.

## 2 Methods

### 2.1 Participants

Participants were recruited from students and employees of urban educational institutions in Hungary through personal solicitation by two researchers. They recruited only those using an iPhone running iOS 12^1^ (released in June 2018) with the built-in *Screen Time* feature, which records the type and duration of application use. Eligibility criteria required participants to be 18 years of age or older and to agree to share their objectively recorded smartphone usage data obtained from Apple's *‘Screen Time’* function for a period of eight consecutive days preceding the study. Given the labor-intensive recruitment process and the requirement to obtain screenshots of iPhone application usage via email, we planned to recruit 100 participants. This sample size was calculated to provide sufficient statistical power for the planned analyses (see *Sample Size Considerations* section below). Therefore, the recruitment followed a sequential non-probability convenience sampling method (Sedgwick, 2013). After obtaining 100 fully completed responses, we concluded the study. Participants' mean age was 24.37 years (SD = 7.78; range = 18–55 years). Considering self-identified gender, 38 participants identified as male and 62 as female. No participants identified themselves as non-binary or other. The study received ethical clearance from the Research Ethics Board at the Faculty of Education and Psychology, ELTE Eötvös Loránd University (Approval No. 0011/2025). All participants provided informed consent before data collection and agreed to the anonymous publication of group results by signing the consent form. The study adhered to the principles of the World Medical Association Declaration of Helsinki (World Medical Association, 2013) for research involving human participants.

### 2.2 Measures

#### 2.2.1 Demographic and iPhone use measures

Demographic variables included age and gender. Participants also rated their perceived overall iPhone use volume on a single four-point ordinal item: 1 (little), 2 (average), 3 (a lot), or 4 (too much). This single-item ordinal rating — referred to throughout as perceived use — was treated as a quasi-continuous variable in the correlation, regression, and mediation analyses, a common practice for ordered categorical predictors; the implications of this treatment are addressed in the Limitations section. Each participant provided screenshots of their iPhone *Screen Time* records covering the preceding eight consecutive days. We used data extracted from the iOS Screen Time feature to quantify total smartphone use, average daily use, and time spent in application usage categories as classified directly by *Screen Time.*

We identified twelve objective application categories: Social Networking, Entertainment, Games, Productivity & Finance, Creativity, Information & Reading, Education, Health & Fitness, Shopping & Food, Travel & Navigation, Utilities, and Other. To support theoretically meaningful analyses and reduce model complexity, application categories were aggregated into two higher-order indices reflecting the dominant motivational functions of smartphone use, following the hedonic–utilitarian distinction established in the technology-use literature (van der Heijden, 2004; Vujić & Szabo, 2022). A *Hedonic Use* category (Appendix) was created by combining Social Networking, Entertainment, Games, Creativity, Shopping & Food, and Travel & Navigation, representing leisure-, pleasure-, and reward-oriented smartphone activity. The *Utilitarian Use* category was formed by combining Productivity & Finance, Information & Reading, Education, Health & Fitness, and Utilities, representing instrumental, goal-directed, and functional smartphone use. We excluded the ‘Other’ category from these composite indices because its content could not be unambiguously classified within either motivational domain. We acknowledge that this category-level assignment cannot capture individual differences in motives (e.g., Creativity applications may serve professional, utilitarian purposes for content creators, while Information & Reading may be leisure-oriented for avid readers); we discuss this partly subjective classification in the Limitations section.

#### 2.2.2 Risk of smartphone addiction (RSA)

Addiction risk was assessed using the Hungarian version (Csibi et al., 2019) of the Smartphone Application-Based Addiction Scale (SABAS; Csibi et al., 2018). The scale consists of six items grounded in the components model of addiction, assessing core addiction-related features such as salience, mood modification, tolerance, withdrawal, conflict, and relapse. Participants indicate their agreement with each statement on a 6-point Likert scale ranging from 1 (strongly disagree) to 6 (strongly agree), with higher scores reflecting a greater RSA. One sample item is: “*Nowadays, my smartphone is the most important part of my everyday life* (salience).” In an adult validation study, the scale demonstrated acceptable internal consistency (Cronbach's alpha [α] = 0.75), and exploratory factor analysis supported a unidimensional structure that accounted for 35% of the total variance. Although the internal consistency of the scale in the current sample was lower (α = 0.65; omega [ω] = 0.77), alpha values ranging between 0.60 and 0.70 are acceptable for exploratory research and brief screening tools (Nunnally & Bernstein, 1994); however, the omega (ω) value was higher. Because the SABAS is a brief screening instrument and does not establish a clinical diagnosis, its scores are interpreted throughout as indices of RSA rather than of clinically validated addiction.

#### 2.2.3 Smartphone deprivation scale

Perceptions of SDF were measured using the Smartphone Deprivation Inventory (Csibi et al., 2019; Pirwani et al., 2025). This instrument comprises nine items designed to assess emotional, psychological, and psychophysiological reactions experienced during prolonged smartphone unavailability. The scale captures symptoms such as irritability, restlessness, nervousness, sadness, sleep disturbance, tension, rigidity, and fatigue. Participants rate how often they experience each symptom when unable to use their smartphone for several hours on a 7-point Likert scale, ranging from 1 (never) to 7 (always). A sample item is: “*When I cannot use my smartphone for a longer period, I feel tense or uneasy*.” In this study, reliability was (α = 0.89; omega [ω] = 0.94). The SDF construct is conceptually adjacent to the withdrawal component of the SABAS; this content overlap is explicitly addressed in the Discussion and Limitations sections.

### 2.3 Statistical analyses

All analyses were conducted using IBM SPSS Statistics (version 29). We assessed scale reliability using Cronbach's alpha and computed descriptive statistics for all variables. We estimated the proportion of participants at high risk of smartphone addiction using the SABAS cut-off score of ≥23 (out of 36), derived by Peng et al. (2023) through latent profile and receiver operating characteristic analyses. Because this cut-off was derived from an adolescent sample and no clinically validated threshold exists for adults, we report the resulting proportion as a descriptive screening estimate rather than a prevalence. Pearson correlation analyses examined associations among RSA, SDF, perceived and objective iPhone use, hedonic and utilitarian application usage time, age, and gender, with component-level relationships visualized using heatmaps. Ninety-five percent confidence intervals (Fisher r-to-z) are reported in the text for the key correlation coefficients. Because the component-level correlations displayed in Fig. 1 are exploratory, they were not adjusted for multiple testing and are interpreted as descriptive patterns rather than confirmatory tests. Application-category usage times were highly positively skewed; they were therefore used descriptively (medians, minima, and maxima are reported in Table 3) rather than as inferential predictors, and results involving the composite hedonic and utilitarian indices should be interpreted with this skew in mind. To identify independent correlates of RSA, we performed a hierarchical multiple regression analysis, entering SDF, hedonic use, and perceived smartphone use in the first step, followed by age and gender in the second step.

**Fig. 1:**
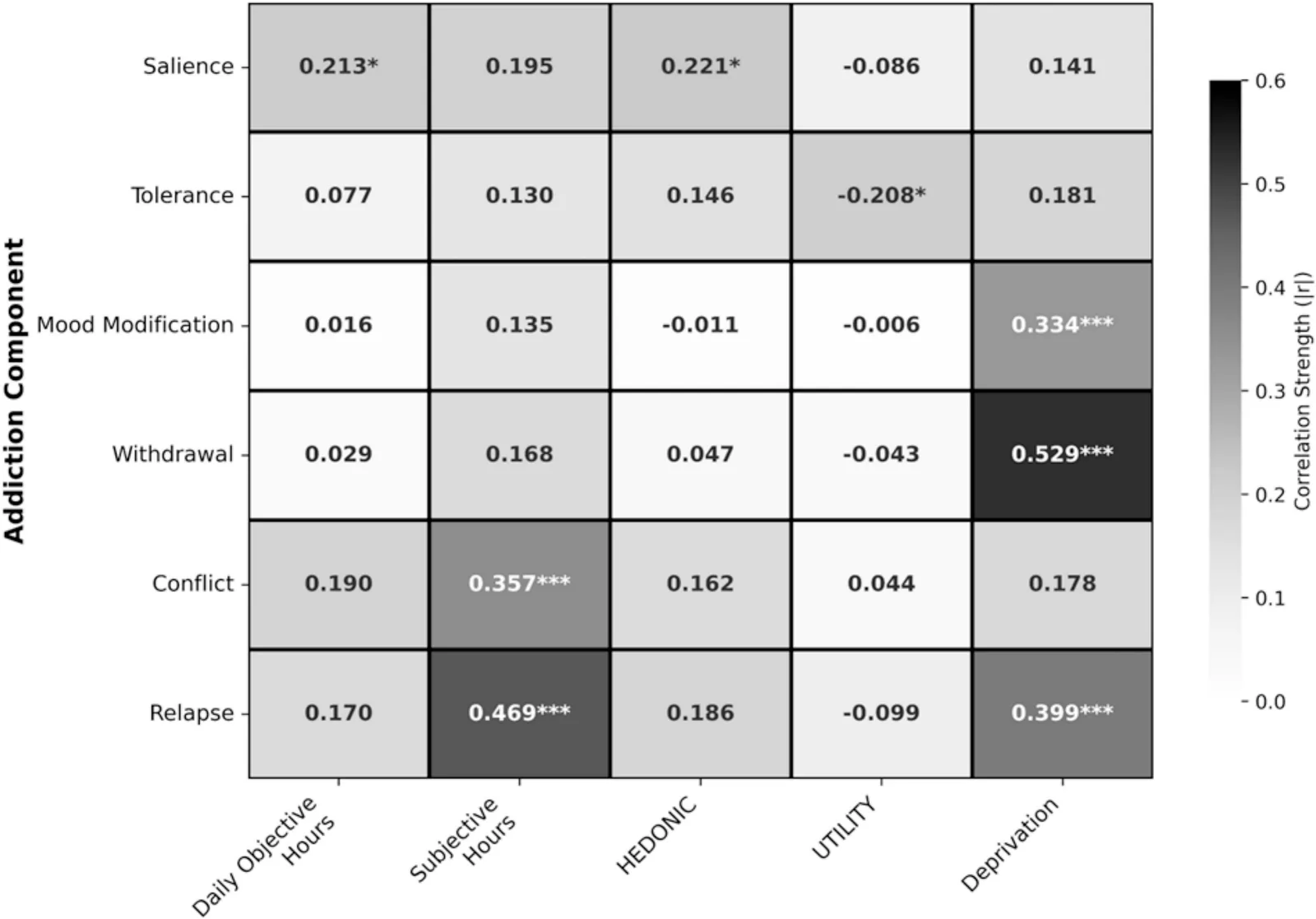
Heatmap of Pearson correlations (r) between addiction components (salience, tolerance, mood modification, withdrawal, conflict, relapse) and usage-related variables (daily objective hours, perceived use, hedonic use, utilitarian use, and deprivation [SDF]). Darker shading indicates stronger associations. Asterisks denote statistically significant correlations (* *p* < .05, ** *p* < .01, *** *p* < .001). Correlations shown are exploratory and unadjusted for multiple testing.

We entered the theoretically motivated psychological and use-related predictors first to test the substantive hypotheses, and the demographic variables second to examine whether they explained incremental variance beyond the substantive predictors. Total objective use was not entered alongside hedonic use because the two indices were nearly collinear (*r* = 0.94; Table 1), making their joint inclusion statistically untenable and their separate inclusion largely redundant; hedonic use was retained on theoretical grounds (Vujić & Szabo, 2022), and the implications of this choice are considered in the Limitations section. In addition, we estimated a path model in which perceived smartphone use was tested as a statistical mediator of the association between objectively logged smartphone use and RSA. We specified the mediation component in advance as a replication of Hitcham et al. (2023), with objective use as the predictor, perceived use as the mediator, and RSA as the outcome (paths: objective use → perceived use, objective use → RSA, and perceived use → RSA). SDF was entered as a second, parallel outcome in the same model, regressed on objective use and perceived use; no directed path between RSA and SDF was specified, and their association was instead represented as a freely estimated residual covariance. These paths describe statistical associations only and carry no directional interpretation. The path model was estimated in JASP 0.19.3 (JASP Team, 2025) using the Mediation Analysis routine of the jaspSem module, which implements lavaan (Rosseel, 2012), with maximum likelihood estimation and perceived use (a four-category ordinal item) treated as quasi-continuous. Data were complete for all four model variables (*N* = 100). Given the cross-sectional design, the model describes patterns of statistical association only, and no causal inference is intended. We report the indirect association and its 95% percentile bootstrap confidence interval (5000 resamples). We additionally report the total association between objective use and RSA. Following Zhao et al. (2010), we describe a significant indirect association with a non-significant direct association as an indirect-only pattern, and we use that terminology in place of the full/partial mediation distinction. We verified assumptions of normality, linearity, homoscedasticity, and multicollinearity before analysis and set statistical significance at *p* < .05. We assessed normality of the regression residuals by visual inspection of normal Q-Q plots in conjunction with the Shapiro-Wilk test.

**Table 1.** Correlations between the study variables.

Measure	1.	2.	3.	4.	5.	6.	7.
1. Age	–						
2. Gender	0.037	–					
3. Risk of Addiction (RSA)	−0.044	0.075	–				
4. Deprivation (SDF)	−0.156	0.059	0.482***	–			
5. Hedonic Use	−0.182	0.047	0.213*	0.109	–		
6. Utilitarian Use #	−0.023	0.099	−0.114	0.083	−0.018	–	
7. Perceived Use ##	−0.222*	0.038	0.406***	0.194	0.333***	0.055	–
8. Objective Use	−0.187	0.076	0.195	0.159	0.942***	0.242*	0.347***

*Note.* *p < .05, **p < .01, ***p < .001. Two-tailed test; #Based on the ‘*Screen Time’* of the iPhone iOS; ## Rated on a single-item 4-point ordinal scale: little, average, a lot, too much. Ninety-five percent confidence intervals for the key coefficients are reported in the text.

### 2.4 Sample size considerations

The primary planned analysis was a regression model of RSA with three substantive predictors (SDF, hedonic use, and perceived use); demographic variables were added in a second, exploratory block. An a priori calculation in G*Power 3.1 (Faul et al., 2009) indicated that *N* = 77 is required to detect a medium effect (Cohen's f^2^ = 0.15) with 80% power at α = 0.05 for the three-predictor model. Thus, the planned sample of *N* = 100 provided adequate power for the primary analysis. For the planned mediation model, Fritz and MacKinnon (2007) recommend approximately 71 participants when both the a and b paths are of medium size — a benchmark exceeded by the corresponding effects reported by Hitcham et al. (2023; a path r = .44–.62; b path r = .38), on which our expectations were based. For two-tailed bivariate correlations (α = 0.05, power = 0.80), *N* = 100 is sufficient to detect effects of r ≈ 0.28 or larger; consequently, smaller correlations (e.g., r ≈ 0.20) could not be reliably detected, and their statistical non-significance is not interpreted in this paper as evidence of their absence.

## 3 Results

### 3.1 Proportion of participants at high risk of smartphone addiction

Based on the SABAS cut-off of ≥23 (Peng et al., 2023), 29 of 100 participants (29.0%, 95% CI [20.6%, 39.1%]) scored in the high-risk range. Because this cut-off was derived in an adolescent sample and the present study used a convenience sample, interpret this figure as a descriptive screening estimate rather than a population prevalence.

### 3.2 Correlations between the types of use, risk of addiction, SDF, gender, and age

RSA was moderately to strongly positively associated with SDF (*r* = 0.48, 95% CI [0.32, 0.62], *p* < .001). RSA was also moderately positively related to hedonic use (*r* = 0.21, 95% CI [0.02, 0.39], *p* < .05). Perceived iPhone use (volume) showed moderate positive associations with RSA (*r* = 0.41, 95% CI [0.23, 0.56], *p* < .001), hedonic use (*r* = 0.33, 95% CI [0.15, 0.50], *p* < .001), and objective iPhone use (daily hours) (*r* = 0.35, 95% CI [0.16, 0.51], *p* < .001). The association between objective use and RSA was weak and did not reach statistical significance (*r* = 0.20, 95% CI [−0.001, 0.38]); given that the study was powered to detect r ≈ 0.28 or larger, this confidence interval is compatible with a small positive association and does not indicate its absence. Objective iPhone use was strongly positively associated with hedonic use (*r* = 0.94, 95% CI [0.92, 0.96], *p* < .001) and weakly with utilitarian use (*r* = 0.24, 95% CI [0.05, 0.42], *p* < .05). Age was weakly inversely related to perceived use (*r* = −0.22, 95% CI [−0.40, −0.03], *p* < .05), but the relationship did not reach statistical significance with objective use. Gender was unrelated to any measure (see Table 1).

### 3.3 Correlation pattern between addiction components and predictor variables

The heatmap reveals distinct patterns of association between addiction components and predictor variables. Withdrawal and mood modification components exhibited the strongest relationships with SDF, consistent with the interpretation that these components reflect psychological dependency processes. In contrast, conflict and relapse showed their strongest associations with perceived use, suggesting these components capture behavioral pattern aspects of RSA. Notably, most components exhibited weak to negligible correlations with objective usage measures (daily objective hours), with salience showing the only significant, albeit modest, relationship. Hedonic use and utilitarian use generally showed minimal associations across components. This differential pattern of correlations is consistent with a multi-pathway model wherein psychological dependency (indexed by withdrawal and mood modification) and habitual behavioral patterns (indexed by relapse and conflict) represent distinct routes to elevated RSA, neither of which appears strongly related to simple usage volume (Fig. 1). These component-level correlations were exploratory and unadjusted for multiple comparisons; the pattern is therefore descriptive and requires confirmatory replication.

### 3.4 Hierarchical regression

Preliminary analyses confirmed that the data met the assumptions for multiple regression. Residual inspection indicated normality (visual inspection of the normal Q-Q plot indicated no substantial departure from the diagonal reference line; Shapiro-Wilk W = 0.978, *p* = .098) and independence (Durbin-Watson = 1.837, *p* = .37). Variance inflation factors ranged from 1.04 to 1.16, indicating no multicollinearity issues. A minor violation of homoscedasticity was detected (Breusch-Pagan χ^2^ = 5.19, *p* = .023); however, this did not substantially affect the interpretation of results, given the robustness of the regression procedure.

The first model (Table 2), including SDF, hedonic use, and perceived use, was statistically significant and accounted for 33.8% of the variance in RSA, R^2^ = 0.338, F(3, 96) = 16.303, *p* < .001. Within this model, SDF emerged as the strongest statistical predictor (β = 0.416, *t* = 4.908, *p* < .001), followed by perceived use (β = 0.302, *t* = 3.384, *p* = .001). However, hedonic use did not significantly predict RSA (β = 0.067, *t* = 0.756, *p* = .452).

**Table 2.** Hierarchical multiple regression analysis predicting risk of smartphone addiction (RSA).

Predictor	B	SE B	95% CI	β	sr^2^	t	p
Constant	8.538	1.723	[5.118, 11.958]	–	–	4.956	<0.001
Deprivation (SDF)	0.278	0.057	[0.166, 0.391]	0.416	0.166	4.908	<0.001
Perceived Use	2.044	0.604	[0.845, 3.243]	0.302	0.079	3.384	0.001
Hedonic Use	0.000	0.000	[−0.001, 0.001]	0.067	0.004	0.756	0.452

*Note.* R^2^ = 0.338, Adjusted R^2^ = 0.317, F(3, 96) = 16.303, *p* < .001; Cohen's f^2^ = 0.509 (large effect size); CI = confidence interval; β = standardized coefficient; sr^2^ = squared semi-partial correlation.

In the second block (Table 2), adding demographic variables (age and gender) did not significantly enhance the model, ΔR^2^ = 0.012, F change (2, 94) = 0.849, *p* = .431. Neither age (β = 0.107, *t* = 1.230, *p* = .222) nor gender (β = 0.030, *t* = 0.360, *p* = .720) contributed unique variance to predicting RSA. Considering the non-significant impact of demographics and the principle of parsimony, the first model without demographic controls is reported as the final model.

A sensitivity analysis re-scoring the SABAS without the withdrawal item (Item 4) yielded a five-item score highly correlated with the full score (*r* = 0.96); SDF remained significantly associated with this reduced score (*r* = 0.39, 95% CI [0.21, 0.54], *p* < .001) and remained a leading predictor in the regression (β = 0.32), indicating that the SDF–RSA association was not attributable to shared withdrawal-item content alone.

### 3.5 Mediation analysis

A mediation analysis was conducted to further contextualize the regression findings. Objective daily iPhone usage was significantly associated with perceived smartphone use (b = 0.116, *p* < .001), and perceived use, in turn, was strongly associated with RSA (b = 2.596, *p* < .001). In contrast, objective smartphone use showed no direct association with RSA (b = 0.139, *p* = .526). The indirect association between objective use and RSA via perceived use was ab = 0.301, 95% percentile bootstrap CI [0.090, 0.613] (5000 resamples), and the total association was b = 0.440, SE = 0.221, z = 1.99, *p* = .047. Because the indirect association was significant while the direct association was not, this pattern is described as indirect-only, following Zhao et al. (2010). The total association was only marginally significant, consistent with the non-significant zero-order correlation between the two variables (*r* = 0.20, 95% CI [−0.001, 0.38]; Table 1), and should therefore be treated as borderline. The indirect path accounted for approximately 68% of the total association, but this ratio is estimated with low precision and is reported for comparability with prior work rather than as a stable effect-size estimate. Neither objective nor perceived use was significantly associated with SDF; however, the residual covariance between RSA and SDF was substantial and statistically significant (cov = 13.85, SE = 3.40, z = 4.08, *p* < .001, 95% CI [7.19, 20.51]), corresponding to a residual correlation of 0.45. Because this term is a covariance rather than a directed path, it describes a symmetric association between the two outcomes and carries no directional interpretation. The indirect association from objective use to SDF via perceived use was not significant (ab = 0.185, *p* = .159), nor was the total association between objective use and SDF (b = 0.536, *p* = .107). The model explained 16.8% of the variance in RSA, 12.0% in perceived use, and 4.7% in SDF. Overall, the pattern is consistent with statistical mediation by perceived use; given the cross-sectional design, however, no causal ordering can be inferred (Fig. 2).

**Fig. 2:**
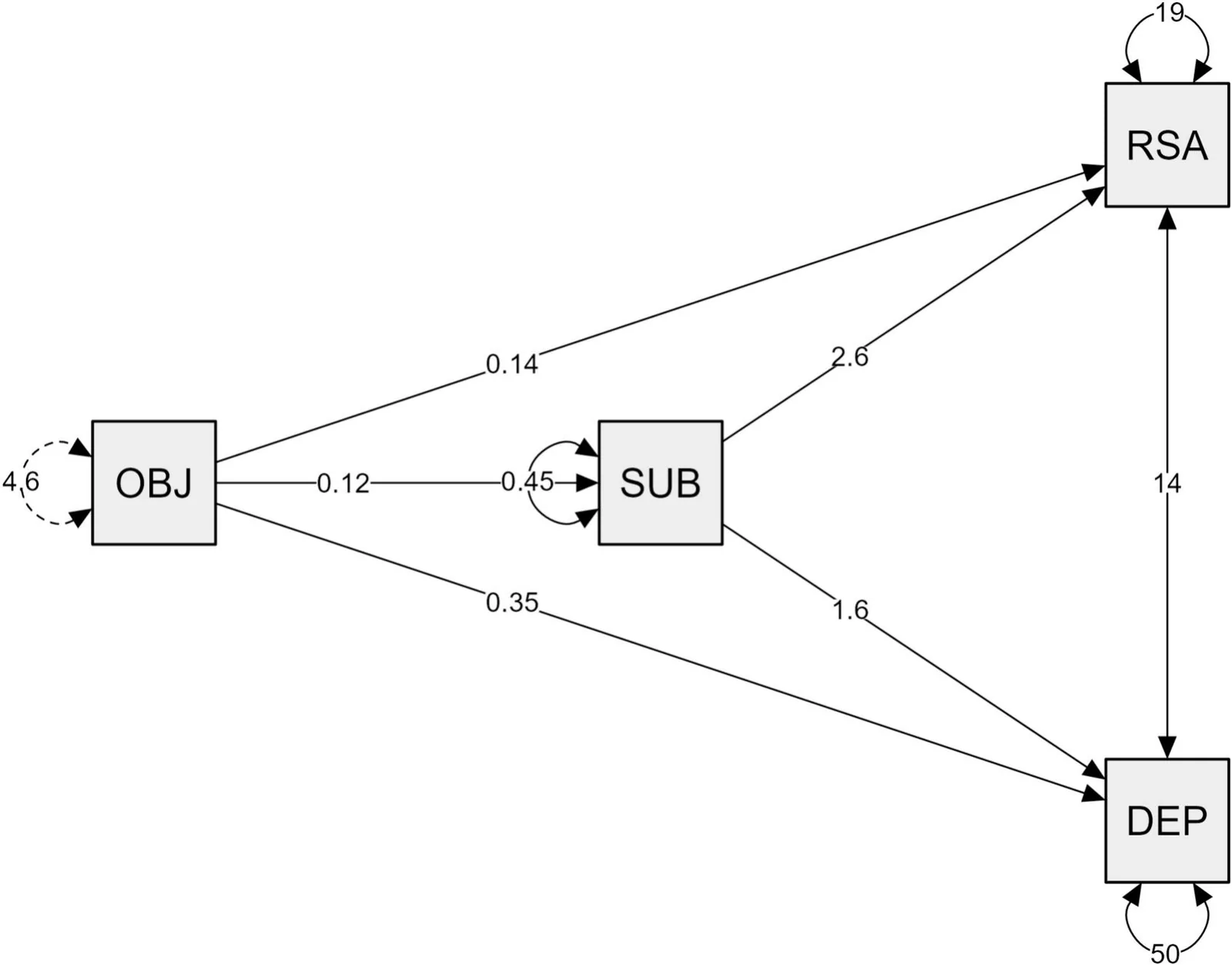
Results of the mediation analysis. *Note:* The diagram shows the unstandardized path coefficients (b-values) estimated by the mediation/structural equation model; OBJ = Objective use/daily minutes; SUB = Perceived use (single-item ordinal rating: little, average, a lot, too much); DEP = deprivation (SDF); RSA = Risk of smartphone addiction. Paths represent statistical associations in a cross-sectional model specified a priori to replicate Hitcham et al. (2023); no causal or directional interpretation is implied. RSA and SDF were modeled as parallel outcomes; the RSA–SDF term is a residual covariance rather than a directed path.

### 3.6 Application usage pattern over eight consecutive days among iPhone users

iPhone use was dominated by social media applications, which accounted for the greatest amount of usage time across the observation period. Entertainment applications ranked second most frequently used, followed by a group of mid-range categories, including Other, Productivity, and Games. Notably, gaming applications showed a highly skewed usage pattern, with low typical use but substantial engagement among a subset of participants. Travel- and creativity-related applications were used to a lesser extent, while utility and shopping applications showed relatively modest engagement. Educational, informational, and health-related applications were the least used categories, with minimal typical usage observed across participants. Overall, the results indicate a strong concentration of smartphone activity in leisure- and social-oriented applications (hedonic use; close to 80%; see Table 3), accompanied by substantial individual differences in usage patterns.

**Table 3.** Descriptive statistics for iPhone usage by category over an 8-Day period.

Rank	Category	Mean (min)	SD (min)	Median (min)	Min	Max
1	***Social media***	1494.72	688.79	1395.50	111	4917
2	***Entertainment***	293.57	458.70	133.50	0	3016
3	Other*	164.07	226.22	86.50	1	1402
4	Productivity	163.55	174.47	120.50	6	1300
5	***Games***	162.37	326.80	2.50	0	1957
6	***Travel***	85.93	130.99	38.00	0	717
7	***Creativity***	76.34	101.28	45.00	2	708
8	Utility	57.45	89.03	15.00	0	469
9	***Shopping***	40.07	49.68	25.00	0	264
10	Education	33.98	124.85	0.00	0	873
11	Information	29.35	49.23	15.50	0	295
12	Health	7.38	23.54	0.00	0	197

*Note. N* = 100. Time values represent total minutes of use across 8 days. Categories ordered by mean usage. High SD indicates substantial individual differences. ***Bold italics*** are apps in the ‘hedonic’ use category. *’Other’ is not listed in the hedonic or utilitarian categories.

## 4 Discussion

The observed proportion of participants scoring above the SABAS screening cut-off (29%) is comparable in magnitude to the global average of 26.99% (95% CI, 22.73–31.73) across 64 countries (Meng et al., 2022), although the two figures are not directly comparable given differences in instruments, cut-offs, and sampling, and the present estimate derives from a convenience sample. The correlational pattern supports recent findings that RSA is more strongly associated with subjective and psychological processes than with objective usage volume (Hitcham et al., 2023). The moderate-to-strong association between RSA and SDF is consistent with research highlighting withdrawal-like experiences and emotional dependence as central to problematic smartphone use (Elhai et al., 2019; Montag et al., 2021). The positive association between RSA and hedonic but not utilitarian use aligns with evidence that reward-oriented, leisure-driven engagement carries greater risk than functional, goal-directed use (Vujić & Szabo, 2022).

An important alternative explanation must be acknowledged. RSA, perceived use, and SDF were all assessed by self-report, whereas objective use was derived from device records. The stronger associations observed among the self-reported variables may therefore partly reflect common method variance and differences in measurement modality rather than genuinely stronger substantive relationships. Moreover, some heavy users may not see their behavior as problematic or may normalize high levels of smartphone use. In a large Danish study, approximately one quarter (26%) of university college students at high risk of smartphone addiction did not identify their smartphone behavior as problematic (Frydenlund et al., 2023). Similarly, Geyer et al. (2021) could not identify reliable differences in logged smartphone activity between self-identified addicted and non-addicted users, and Bakar Kahraman and Yeni Elbay (2026) found that objective screen time and addiction symptoms captured partly non-overlapping groups of risky smartphone users. Accordingly, the weak association between objective use and RSA observed here should not be interpreted as direct evidence that objective use is unimportant: the study was powered to detect correlations of r ≈ 0.28 or larger, and the confidence interval around the observed coefficient (*r* = 0.20, 95% CI [−0.001, 0.38]) is compatible with a small positive relationship. Our conclusions are therefore restricted to the relative primacy of subjective appraisal within this design.

Perceived smartphone use showed consistent associations with RSA, hedonic use, and objective use, whereas objective use exhibited only weak links with RSA, reinforcing that subjectively perceived use relates to RSA more closely than logged screen time (Hitcham et al., 2023). The weak inverse relationship between age and perceived use, coupled with the absence of gender effects, suggests that RSA is associated less with demographic factors than with how individuals experience and interpret their engagement with the device. The absence of demographic effects is consistent with the mixed picture in the literature: although some studies report higher problematic use among females or younger users, systematic reviews indicate that demographic associations are inconsistent and generally small once psychological factors are considered (Busch & McCarthy, 2021; Meng et al., 2022; Pirwani et al., 2025). Overall, these results align with recent literature advocating a shift from volumetric models to psychologically focused frameworks that emphasize psychological dependence and motivational orientation (James et al., 2023).

Component-level correlations revealed distinct descriptive patterns of association with RSA (Fig. 1). Withdrawal and mood modification showed the strongest relationships with SDF, consistent with the view that these components reflect psychological dependency rather than behavioral excess. This pattern aligns with experimental evidence that smartphone restriction provokes psychological withdrawal symptoms (Eide et al., 2018). It also supports theoretical models positioning perceived loss of control and psychological vulnerability as central to the RSA (Billieux et al., 2015). Conversely, conflict and relapse showed moderate-to-strong associations with perceived use, suggesting these components capture habitual behavioral dysregulation. This dissociation is consistent with pathway models proposing that problematic smartphone use emerges through multiple routes (Billieux, 2012), with some individuals experiencing primarily psychological symptoms, others predominantly behavioral patterns. The stronger association of conflict and relapse symptoms with perceived rather than actual iPhone use corroborates findings that RSA is more closely tied to metacognitive beliefs about one's behavior (Hitcham et al., 2023).

Further consideration concerns the conceptual overlap between the SDF measure and the SABAS. The SDF instrument assesses withdrawal-like reactions to smartphone unavailability, and the SABAS itself contains a withdrawal item; indeed, the withdrawal component showed the strongest correlation with SDF in the component-level analysis (Fig. 1). The emergence of SDF as the strongest statistical predictor of RSA may therefore reflect, at least in part, shared construct content rather than an independent psychological process, and the SDF–RSA association should be regarded as an upper-bound estimate that likely includes overlap-driven variance. To probe this overlap, we conducted a sensitivity analysis re-scoring the SABAS with the withdrawal item (Item 4) removed.^2^ The resulting five-item score correlated very highly with the full six-item score, and its association with SDF remained substantial and statistically significant. In the regression model, SDF remained a leading predictor of the reduced score (β = 0.32), comparable in magnitude to perceived use (β = 0.32). The SDF–RSA association therefore does not appear to be an artifact of shared withdrawal-item content alone, although some conceptual overlap remains and the tempered interpretation above still holds. Notably, SDF also correlated substantially with the mood-modification component, suggesting that item overlap is unlikely to fully account for the association; nevertheless, future studies using longer, multidimensional RSA instruments should test whether deprivation feelings relate to non-withdrawal addiction components when withdrawal content is held constant.

Notably, all addiction components showed weak to negligible correlations with objective usage measures, with only salience demonstrating modest significance. This finding questions strictly volume-based conceptualizations and extends research demonstrating weak associations between logged smartphone use and addiction severity (Hitcham et al., 2023; Parry et al., 2021), while remaining compatible with small volume–RSA associations that this study was not powered to detect. The limited incremental predictive value of objective usage suggests that RSA, as captured by brief screening scales, may reflect subjective psychological distress and perceived loss of control rather than quantifiable behavioral excess (Davidson et al., 2022).

Negligible associations between hedonic and utilitarian categories and most components further indicate that content type alone does not account for addiction symptoms. This differential pattern is consistent with a multi-pathway model in which psychological dependence and behavioral dysregulation represent distinct routes to elevated RSA, neither of which is strongly determined by usage volume. Theoretically, this dissociation can be situated within the Interaction of Person-Affect-Cognition-Execution (I-PACE) framework (Brand et al., 2016), in which problematic use emerges from the interplay of affective processes (here indexed by deprivation sensitivity and mood modification), cognitive processes (metacognitive appraisal of one's own use, indexed by perceived use), and executive/behavioral processes (habitual dysregulation, indexed by conflict and relapse), rather than from exposure time per se. On this account, subjective appraisal and affective reactivity to unavailability are proximal correlates of problematic engagement, whereas raw usage volume is a distal input whose effect depends on appraisal. This framing clarifies why self-referential measures (perceived use, SDF) track RSA scores more closely than logged time and generates the testable prediction that appraisal and affective reactivity should mediate any volume–risk association in longitudinal designs.

Hierarchical regression indicated that SDF and perceived use were independently associated with RSA, whereas objective hedonic use and demographic variables contributed no unique variance. This pattern aligns with recent evidence that RSA is more closely related to subjective appraisal and psychological dependence than objectively logged time (Hitcham et al., 2023). A large meta-analysis revealed only modest correspondence between self-reported and objectively measured digital media use, suggesting that problematic-use scales capture metacognitive beliefs and distress rather than behavioral volume per se (Parry et al., 2021). The strong statistical role of SDF supports experimental findings that smartphone restriction elicits withdrawal-like symptoms, indicating RSA may reflect psychological dependency processes rather than mere exposure time (Eide et al., 2018). These findings reinforce a shift from volumetric models toward frameworks emphasizing perceived loss of control and SDF sensitivity as instrumental RSA features.

The mediation analysis further contextualized the regression findings. Consistent with regression results showing no direct predictive value of objective smartphone use, objective smartphone use was associated with RSA only indirectly, via perceived use, in the statistical model. Given the cross-sectional design, this pattern reflects statistical mediation only and cannot establish temporal or causal ordering; the directional paths in Fig. 2 reflect the a priori model specification adopted to replicate Hitcham et al. (2023), not demonstrated causal processes. This pattern is consistent with prior findings by Hitcham et al. (2023), who reported that perceived use accounted for most associations between logged screen time and RSA across two time points. The present study constitutes a more limited replication, however: it is cross-sectional rather than two-wave, draws on a smaller sample, and defines the mediator as a single four-category ordinal item rather than a continuous usage estimate. The present results extend Hitcham et al.'s results by revealing that neither objective nor subjective use was significantly associated with SDF; instead, RSA and SDF showed a substantial residual covariance — a symmetric, non-directional association that is nonetheless consistent with experimental evidence that smartphone restriction elicits withdrawal-like psychological symptoms central to addictive behavior (Eide et al., 2018). The regression and mediation findings converge on a coherent interpretation. Scores on brief RSA screening instruments appear to reflect subjective appraisal of one's use and sensitivity to deprivation more than objectively quantifiable screen time. This interpretation aligns with broader evidence of limited correspondence between logged digital behavior and self-reported problematic use (Parry et al., 2021). It also reinforces calls to complement volumetric models with psychologically grounded frameworks of behavioral addiction.

Screen Time categorized over 50% of total objective iPhone use as social media. Including this category, approximately 80% of total objective use could be classified as hedonic (see Table 3). Contrary to theoretical expectations, hedonic use contributed only modestly to RSA. While Vujić and Szabo (2022) found hedonic smartphone use to be a significant predictor in their structural equation model, our findings suggest a more nuanced relationship. This discrepancy may reflect methodological differences or indicate that the relationship between pleasure-seeking motives and RSA is moderated by factors not captured in bivariate associations. Furthermore, Vujić and Szabo assessed hedonic use based on subjective reports, unlike in the current study, and — as noted above — the assignment of application categories to motivational domains is itself an approximation of users' actual motives.

### 4.1 Practical and clinical implications

Given the caveats that the design was cross-sectional, the sample was non-clinical, and RSA was assessed with a brief screening scale, several tentative implications follow. First, screening and assessment for at-risk smartphone use should incorporate perceived use and deprivation-related experiences alongside, not instead of, objective indicators, because self-appraisal and reactivity to unavailability tracked RSA scores more closely than logged time. Second, interventions that focus exclusively on reducing screen time (e.g., time-limit apps or digital-detox regimens based on displayed usage statistics) may miss the subjective processes most closely tied to risk; cognitive-behavioral and metacognitive approaches that target beliefs about one's own use, tolerance of device unavailability, and mood-regulation alternatives may be better aligned with the correlates identified here and in prior work (Billieux et al., 2015; Brand et al., 2016). Third, for individuals reporting strong feelings of deprivation, graduated exposure to phone-free periods combined with coping-skills training could be piloted and evaluated. These suggestions are hypotheses for intervention research, not clinically validated recommendations.

### 4.2 Limitations

This study has several limitations that should be considered when interpreting the results. First, the cross-sectional design prevents causal interpretation of observed regression and mediation pathways, and the directional paths in the mediation model reflect specification choices rather than established temporal ordering. Second, despite documented statistical power for medium-sized effects, the modest sample size and convenience-based recruitment limit generalizability, as does the sample's cultural homogeneity; in particular, the study could not reliably detect small associations (*r* < 0.28), so weak effects of objective use cannot be ruled out. Third, exclusive reliance on iPhone (iOS) users restricts applicability to other smartphone platforms with different usage patterns and logging systems. Fourth, RSA, perceived use, and SDF were all self-reported, whereas objective use was device-logged; the stronger associations among the self-reported variables may therefore be inflated by common method variance (Frydenlund et al., 2023; Geyer et al., 2021). Fifth, the SDF measure and the SABAS share withdrawal-related content, so the SDF–RSA association is likely a modest upper-bound estimate; however, a sensitivity analysis removing the withdrawal item (see Discussion) indicated that the association is robust to withdrawal-item overlap. Sixth, RSA was assessed with a brief six-item screening scale using a cut-off derived in adolescents (Peng et al., 2023) rather than a clinical evaluation; the reported high-risk proportion is a screening estimate, not a diagnosis-based prevalence. Seventh, perceived use was measured with a single four-category ordinal item treated as quasi-continuous, which constrains its variance and precision. Eighth, the assignment of Screen Time categories to hedonic and utilitarian indices, although grounded in prior conceptualizations (van der Heijden, 2004; Vujić & Szabo, 2022), could not account for individual differences in motives (e.g., Creativity may be utilitarian for professional content creators, and Information & Reading may be hedonic for avid readers) and is therefore partly subjective. Ninth, the component-level correlations were exploratory and unadjusted for multiple testing. Finally, future research should employ ecological momentary assessment to capture real-time, within-person links between use episodes, perceived use, and deprivation feelings, and should use multidimensional RSA instruments with clinically anchored thresholds.

## 5 Conclusion

This study provides empirical support for applying Griffiths' (2005) components model to RSA research while highlighting the particular importance of withdrawal-related experiences (SDF). Within the limits of a cross-sectional design and a brief self-report screening instrument, the findings indicate that questionnaire-assessed RSA is more closely associated with how individuals perceive their smartphone use, and how they react to its unavailability, than with objectively logged use volume; they do not establish clinically validated addiction, nor do they show that objective use is irrelevant. The study contributes to addressing current problems in the field in three concrete ways. First, it demonstrates the feasibility of combining freely available, operating-system-level objective records with motivational (hedonic vs. utilitarian) categorization, offering a low-burden template for reducing the field's reliance on self-reported use volume. Second, it indicates that assessment of at-risk use should incorporate perceived use and deprivation feelings alongside objective indicators, helping to explain why usage volume alone identifies at-risk individuals poorly. Third, it implies that interventions and digital well-being tools relying solely on displayed usage time may miss the subjective processes most closely tied to risk, pointing them instead toward appraisal- and deprivation-focused targets. As smartphones become increasingly embedded in daily life, distinguishing genuinely problematic patterns from adaptive, high-engagement behaviors remains a critical challenge for both research and clinical practice.

## Declaration of AI use

The authors used ChatGPT 5.2 and Claude AI (Anthropic, v. Opus 4.8) for proofreading, grammatical verification, completeness checks, and assistance with revisions. They assume full responsibility for the report's contents.

## Ethics and consent

The research was approved by the Research Ethics Board at the Faculty of Education and Psychology, Eötvös Loránd University (Approval No. 0011/2025). All participants signed a consent form and agreed to the publication of the anonymous group results.

## CRediT authorship contribution statement

**Attila Szabo:** Writing – original draft, Supervision, Methodology, Formal analysis, Conceptualization. **Róbert Járai:** Writing – review & editing, Methodology, Formal analysis. **Norbert Tabi:** Resources, Investigation, Data curation. **Dorina Montvai:** Writing – review & editing, Investigation, Data curation. **Celal Bulgay:** Writing – review & editing, Validation. **Robert Podstawski:** Writing – review & editing, Conceptualization. **Angéla Somogyi:** Visualization, Validation, Supervision.

## Funding

This research did not receive financial support.

## Declaration of competing interest

The authors declare that they have no known competing financial interests or personal relationships that could have appeared to influence the work reported in this paper.

## Data Availability

The data are available in the Mendeley Data repository (DOI: 10.17632/kgccp7548m.1)

## Appendix A Appendix

Based on Apple iOS Screen Time's standard application classification, the analyses used the following 12 application usage categories (as provided directly by Screen Time, without researcher reclassification): **Bold** apps = hedonic use; non-bold apps = utilitarian use; Other = uncategorized. The assignment follows the hedonic–utilitarian distinction of van der Heijden (2004) as applied to smartphones by Vujić and Szabo (2022); the manuscript discusses its limitations.

1. **Social Networking – social media and communication platforms (e.g., social feeds, messaging-based social apps).**
2. **Entertainment – video and music streaming, media consumption apps.**
3. **Games – all gaming applications.**
4. Productivity & Finance – work-related, organizational, and financial management apps.
5. **Creativity – photo, video, music creation, and editing applications.**
6. Information & Reading – news, books, reference, and informational content.
7. Education – learning, language, and educational support apps.
8. Health & Fitness – physical activity, health monitoring, and well-being apps.
9. **Shopping & Food – online shopping, food delivery, and retail-related apps.**
10. **Travel & Navigation – maps, navigation, transportation, and travel planning apps.**
11. Utilities – system tools, settings, and functional utility apps.
12. Other – applications not classified into the above categories by Screen Time.

## Footnotes

1 To ensure consistency in logging algorithms and application categorization, the study was limited to Apple iOS *Screen Time* data; hence, iPhone users only.

2 The withdrawal item was removed to test whether the SDF–RSA association reflected shared item content. Because this departs from the validated unidimensional six-item SABAS structure and reduces comparability with the SABAS literature, the five-item score (Cronbach's α = 0.61) is reported only for this sensitivity check and is not used in any other analysis.
